# Isolation and Identification of an Endophytic Fungus *Aspergillus* sp. and Its Growth-Promoting Effects on *Nymphaea candida* Seedlings Through Modulation of the Rhizosphere Microbial Community

**DOI:** 10.3390/microorganisms14050993

**Published:** 2026-04-28

**Authors:** Yuwei Xing, Jingru Zhang, Cong Liu, Yang Liu, Jun Wang

**Affiliations:** College of Marine Life Sciences, Ocean University of China, Qingdao 266003, China; 15335362168@163.com (Y.X.); zhangjingru_66@163.com (J.Z.); liucong6899@163.com (C.L.)

**Keywords:** *Aspergillus* sp., *Nymphaea candida* Presl, endangered plant propagation, photosynthetic pathway

## Abstract

*Nymphaea candida* Presl is a rare and endangered waterlily species, and cultivating robust seedlings suitable for artificial propagation has become a critical issue for the conservation of this species. In this study, *Aspergillus* sp., an endophytic fungus isolated from the roots of *N. candida*, showed the capability of solubilizing phosphate and potassium and producing siderophores. The application of *Aspergillus* sp. significantly increased leaf length, leaf width, leaf number, and root length of *N. candida* seedlings by 97.83%, 131.37%, 94.12%, and 171.25%, respectively. Meanwhile, *Aspergillus* sp. application significantly enhanced soil organic matter content, alkali-hydrolyzable nitrogen content, sucrase activity, and peroxidase activity by 6.57%, 31.62%, 23.26%, and 7.53%, respectively. Moreover, *Aspergillus* sp. enriched beneficial microorganisms including *Cyanobium*, *Aquicella*, and *Cryptomycota* to form a more stable rhizosphere soil microenvironment. Additionally, *Aspergillus* sp. upregulated genes involved in photosynthesis and photosynthesis–antenna protein pathways in *N. candida* leaves, with the expression levels of *psbA*, *petG*, and *psbH* significantly increasing by 2.17, 4.48, and 0.28-fold, respectively. Therefore, the endophytic fungus *Aspergillus* sp. might be a reliable tool for the propagation of *N. candida* seedlings, which would be helpful for the conservation of this rare and endangered aquatic plant species.

## 1. Introduction

The genus *Nymphaea* comprises approximately 50 species of perennial aquatic plants globally, with 5 native species in China [1]. Among them, *Nymphaea candida* Presl, a rare and endangered species, is found exclusively in the Xinjiang region, China. *N. candida* plays an essential role in water purification, habitat provision for wildlife, and maintenance of the structural and functional integrity of wetland ecosystems [2,3]. Over the past decade, the natural distribution area of *N. candida* population has been continuously declining due to the heavy seed predation by waterfowl, its inherently low natural germination rate, and the susceptibility to aquatic diseases during the germination process [4,5]. Currently, *N. candida* has been listed on both the IUCN Red List of Threatened Species and the National Key Protected Wild Plants List of China [6,7]. Given that natural reproduction is insufficient for the effective conservation of *N. candida*, it is urgent to conduct artificial seedling breeding of *N. candida*. Thus, how to promote the early growth of *N. candida* and obtain robust seedlings has become a critical issue for the conservation of this endangered species.

Plant growth-promoting bacteria could significantly promote the growth of aquatic plants due to their capabilities of nitrogen fixation, phosphate solubilization, and phytohormone secretion [8,9,10]. For instance, *Bacillus subtilis* and *Pseudomonas fluorescens* increased the fresh weight of hydroponically cultivated lettuce and celery by 144.4% and 300.9%, respectively [11]. *Bacillus velezensis* significantly increased the dry weight of rice (*Oryza sativa*) by 20.47% [12]. These bacteria could accelerate root development and enhance plant growth primarily by secreting growth hormones [13,14]. Compared with plant growth-promoting bacteria, the potential of plant growth-promoting fungi—particularly endophytic ones—to enhance hydrophyte growth has been substantially underestimated. Endophytic growth-promoting fungi, a group of fungi capable of colonizing internal plant tissues, could promote plant growth, enhance stress resistance, and inhibit pathogenic microorganisms [15]. The most extensively studied endophytic growth-promoting fungi primarily include species from the genera *Trichoderma*, *Penicillium*, and *Talaromyces*, with most studies focusing on their growth-promoting effect for terrestrial plants [16,17,18,19]. For example, *Penicillium commune* EP-5 significantly increased the root length of maize (*Zea mays*) by 39.87% through the production of indole-3-acetic acid (IAA) [20]. *Aspergillus* spp. enhanced the root growth in cassava (*Manihot esculenta*) by increasing soil nutrient availability via phosphate solubilization [21]. In addition, *Trichoderma harzianum* and *Metarhizium anisopliae* were reported to increase the fresh weight of water lettuce (*Pistia stratiotes*) by 23.33% and 10.3%, respectively [22]. We hypothesized that endophytic growth-promoting fungi isolated from *N. candida* might promote seedling growth and thereby provide a reliable tool for artificial propagation of *N. candida* seedlings.

In this study, endophytic fungi were firstly isolated and identified from the roots of *N. candida*. Their taxonomic status was determined using a combination of morphological and molecular biological methods, and their growth-promoting potential was also evaluated. Subsequently, the selected endophytic fungal strain was inoculated into a cultivation system of *N. candida* seedlings, and its effects on seedling growth were assessed by periodically measuring growth parameters, including leaf length, leaf width, leaf area, and root length. Furthermore, the changes in the soil physicochemical properties and enzyme activities of the rhizosphere soil of *N. candida* seedlings were measured to evaluate the impacts of the endophytic growth-promoting fungus on soil nutrient composition. Finally, high-throughput sequencing technologies were employed to analyze the effects of the endophytic growth-promoting fungus on the rhizosphere microbial community structure and the leaf transcriptome of *N. candida* seedlings to elucidate the potential growth-promoting mechanisms. The findings of this study would provide technical support for the seedling cultivation of *N. candida*.

## 2. Materials and Methods

### 2.1. Biological Materials and Reagents

*N. candida* plants were collected from Bosten Lake in the Xinjiang Uygur Autonomous Region, China, on 25 April 2024. Potato Dextrose Agar (PDA) medium used for the isolation of endophytic fungi, and Pikovskaya’s Agar Medium (PVK), Phosphate-solubilizing bacteria medium (OPM), Potassium bacteria Medium (PSM), and Chrome Azurol S Agar Medium (CAS) used for performance characterization, were purchased from Aladdin Biochemical Technology Co., Ltd. (Shanghai, China).

### 2.2. Isolation, Purification, and Performance Characterization of Endophytic Fungi

After transport to the laboratory, roots (5–6 cm in length) were excised from *N. candida* plants and rinsed with tap water for 5–10 min to remove surface debris. The roots were then cut into approximately 1 cm segments. Surface sterilization was performed by sequentially immersing the segments in 75% ethanol for 10 s, rinsing with sterile water, soaking in a 0.1% mercuric chloride solution for 12 min, and finally rinsing 5 times with sterile water [23]. The sterilized tissue segments were inoculated onto PDA medium in the dark at 28 °C for 3–5 days. Once the mycelia emerged around the tissue segments, a mycelial plug (approximately 5 mm in diameter) was excised using an inoculation needle and placed agar side down in the center of a fresh PDA plate for purification. This procedure was repeated until pure cultures were obtained, and the purified strains were streaked onto PDA slants. After the slants were fully covered with mycelia, they were temporarily stored at 4 °C for subsequent spore collection [24]. In addition, mycelial plugs (5 mm diameter) of each strain were inoculated onto the center of PVK, OPM, PSM, and CAS plates to assess the growth-promoting potential. These plates were incubated in the dark at 28 °C for 3–5 days, and the growth status of each strain on the respective functional media was observed.

### 2.3. Molecular Identification and Phylogenetic Tree Construction of Endophytic Fungi

Purified endophytic fungal strains were inoculated onto PDA plates and incubated at 28 °C for 48–96 h to observe and photograph colony morphology [25]. Subsequently, the purified endophytic fungal hyphae were ground with sterile glass beads, and the genomic DNA was extracted using Invitrogen Genomic DNA Extraction Kits (Invitrogen Corporation Shanghai Representative Office, Shanghai, China). NS1 (5′-GTAGTCATATGCTTGTCTC-3′)/NS6 (5′-GCATCACAGACCTGTTATTGCCTC-3′) primers were used for PCR amplification, and PCR products were analyzed by 1% agarose gel electrophoresis. The target band was recovered and purified using a purification kit, and the purified PCR products were sent for Sanger sequencing by Sangon Biotech Co., Ltd. (Shanghai, China). The obtained sequences were subjected to blast sequence homology comparison (https://blast.ncbi.nlm.nih.gov/Blast.cgi, accessed on 7 May 2025) with other fungal sequences in the National Center for Biotechnology Information (NCBI) database with a similarity greater than 99% [26]. Multiple sequence alignments of the tested strains and related reference strains were performed using MEGA software (version 7.0.14), and phylogenetic trees were constructed using the neighbor-joining method [27].

### 2.4. Preparation of Endophytic Fungal Inoculum

The purified endophytic fungal strain was inoculated onto PDA plates and incubated in the dark at 28 °C for 7 days until the plates were fully covered with mycelia. The mycelia were then scraped off using a sterile inoculation spoon, rinsed with sterile water, and filtered through two layers of gauze to remove mycelial fragments. Spores were collected from the filtrate, counted using a hemocytometer, adjusted to 1 × 10^8^ spores/mL with sterile water, and used as the inoculum in subsequent experiments [28].

### 2.5. Growth-Promoting Effect of Endophytic Fungi on N. candida Seedlings

A 60-day pot experiment was conducted starting on 30 May 2025 to investigate the effects of endophytic growth-promoting fungi on the growth of *N. candida* seedlings. Environmental conditions during the experiment: temperature was maintained at 28 ± 2 °C, relative humidity at 60 ± 5%, and light intensity at 200 μmol m^−2^ s^−1^ with a 14/10 h light/dark photoperiod. The fungal strain used was *Aspergillus* sp., a potential endophytic growth-promoting fungus isolated from the roots of *N. candida.* Two groups were established: a control group (CK) and an *Aspergillus* sp. group. Each group contained three replicates, and each replicate consisted of 6 pots. Planting soil (a 1:1 mixture of peat and silt) was sterilized by high-temperature autoclaving and evenly distributed into 36 plastic planting boxes (30 cm × 20 cm × 12 cm), with each box filled to a depth of 8 cm. The boxes were placed in standing water for 48 h and then randomly divided into two groups. For the *Aspergillus* sp. group, *N. candida* seedlings at the two-leaf stage with uniform growth were randomly selected, and their roots were immersed in *Aspergillus* sp. inoculum for 20 min. Then, the seedlings were fixed in the planting boxes by inserting approximately 5 cm of the rhizome apex into the planting soil. Six planting boxes were placed in each glass tank, and the positions of the planting boxes were randomly changed daily to minimize errors caused by variations in light intensity. On the 7th, 14th, and 21st days post-transplantation, 5 mL of the *Aspergillus* sp. spore suspension (1 × 10^8^ spores/mL) was injected into the water of the *Aspergillus* sp. group using a sterile syringe. The control group was immersed in sterile water on the 1st day and received an equal volume of sterile water on the 7th, 14th, and 21st days post-transplantation. Leaf length, leaf width, and leaf number of *N. candida* were measured on the 14th, 28th, 42nd, and 60th days post-transplantation.

### 2.6. Sample Collection

Sixty days post-transplantation, soil samples were collected from the control and *Aspergillus* sp. groups. Intact root–soil complexes were carefully excavated by vertically digging along the edge of each plastic planting box to the bottom using a sterile shovel. Loosely adhering soil was gently shaken off. Rhizosphere soil, defined as the soil tightly adhering to the roots, was collected using a sterile brush and stored at −80 °C for 18S rRNA and 16S rRNA gene sequencing. Meanwhile, bulk soil from each planting box was collected, air-dried, and stored to determine the soil physicochemical properties. Root length was measured using a ruler, and the *N. candida* plants after soil removal were placed in sterile sampling bags and stored at −80 °C for subsequent leaf RNA extraction and transcriptome sequencing [29].

### 2.7. Determination of Soil Physicochemical Properties

Air-dried soil samples from each planting box were ground and passed through 10- and 100-mesh sieves [30]. The contents of alkali-hydrolyzable nitrogen (AN), soil organic matter (SOM), soil sucrose (SC) and peroxidase activities (POD) of the soil were determined using the alkaline diffusion method [31], potassium dichromate (K_2_Cr_2_O_7_) volumetric method with dilution heat [32], and specific assay kits (Solarbio, Beijing, China), respectively.

### 2.8. Rhizosphere Soil DNA Extraction and High-Throughput Sequencing

Total DNA was extracted from rhizosphere soil samples using the FastDNA SPIN Kit for Soil (MP Biomedicals, Solon, OH, USA), and the extracted genomic DNA was examined by electrophoresis on a 1% agarose gel. PCR amplification was performed using universal primers for the 16S rRNA gene (27F-1492R: 5′-AGRGTTYGATYMTGGCTCAG-3′ and 5′-RGYTACCTTGTTACGACTT-3′) and the 18S rRNA gene (EukA-EukB: 5′-AACCTGGTTGCTGCCAGTGCAGT-3′ and 5′-TGATCCTTGCAGGTTCACCTAC-3′) [33]. The PCR program consisted of an initial denaturation at 98 °C for 30 s; 32 cycles of denaturation at 98 °C for 10 s, annealing at 54 °C for 30 s, and extension at 72 °C for 45 s; and a final extension at 72 °C for 10 min. PCR products were pooled and detected by 2% agarose gel electrophoresis, and target bands were excised and purified using the AxyPrepDNA Gel Extraction Kit (CA, USA). Purified products were eluted with Tris-HCl and checked by 2% agarose electrophoresis [34]. Paired-end sequencing was performed on a PacBio platform at Shanghai Lingen Biotechnology Co., Ltd. (Shanghai, China). High-fidelity (hifi) reads were generated using SMRT Link Analysis software (version 11.0). Barcode-CCS sequence identification was performed using Lima (version 2.6.99). Raw read data were trimmed and quality-controlled using QIIME2, and chimeric sequences were removed using DADA2. Representative amplicon sequence variants (ASVs) were assigned to the SILVA (version 13.8) or UNITE (version 8.0) database.

### 2.9. N. candida Leaf RNA Extraction and Transcriptome Sequencing

Healthy *N. candida* leaves (*n* = 6) with similar size were collected from the control and *Aspergillus* sp. groups, rinsed with sterile water, freeze-dried, and immediately ground. RNA was extracted from the disrupted cells using a TRIzol reagent kit (Thermo Fisher Scientific, Waltham, MA, USA). The quantity and quality of the isolated RNA samples were assessed using an Agilent 2100 Bioanalyzer (Agilent Technologies, Santa Clara, CA, USA) and agarose gel electrophoresis to check for RNA degradation. Upon quality approval, the samples were sent to Shanghai Majorbio Bio-pharm Technology Co., Ltd. (Shanghai, China) for sequencing on a NovaSeq X Plus platform. Following sequencing, de novo assembly of clean data from all samples was performed using Trinity as described by Sharma et al. [35]. The assembly results were optimized, evaluated, and subjected to expression level analysis. Based on the quantification results of expression levels, differential gene expression analysis was conducted between the two groups to identify differentially expressed genes (DEGs). DESeq2 software (v 1.46.0) was used for this analysis, with a screening threshold of |log_2_FC| ≥ 1 and *p* < 0.05.

### 2.10. Statistical Analysis

Data visualization was finalized using Origin 2019 software to ensure the standardization and readability of the figures. All data were first tested for normality using the Shapiro–Wilk test and for homogeneity of variances using Levene’s test. For normally distributed data with equal variances, a two-tailed Student’s *t*-test was used for comparisons between two groups. For data that did not meet normality or homogeneity assumptions, the Mann–Whitney U test was applied. Statistical significance was set at *p* < 0.05. Statistical analyses were performed using IBM SPSS Statistics 22 software, and permutational multivariate analysis of variance (PERMANOVA) was employed to analyze the differences in community structure. Differences in ASV relative abundance were compared using the DESeq2 package in R software (v 0.5.0). ASVs with *p* < 0.05 (FDR-adjusted) and |log_2_ (fold change)| > 2 were considered significantly different. Graphing was performed using GraphPad Prism (version 9.0.0.121). The R software tool (version 3.3.1) was used to count the number of common and unique ASVs across all experimental groups. Redundancy analysis (RDA) and associated plotting were performed using the vegan package (version 2.4.3) in R software. Expression levels for each transcript were calculated based on the transcripts per million reads (TPM) method to identify differentially expressed genes (DEGs) between the two sample groups. Cluster analysis was conducted using the Uparse algorithm (Uparse v7.0.1001) on all Effective Tags from all samples. Gene abundance was quantified using RSEM (v 1.3.3), and differential expression analysis was conducted with DESeq2 (v 1.46.0). KEGG pathway enrichment analysis was performed using the Python scipy package (v 1.17.0).

## 3. Results

### 3.1. Isolation and Identification of Endophytic Fungi from N. candida

**Figure 1:**
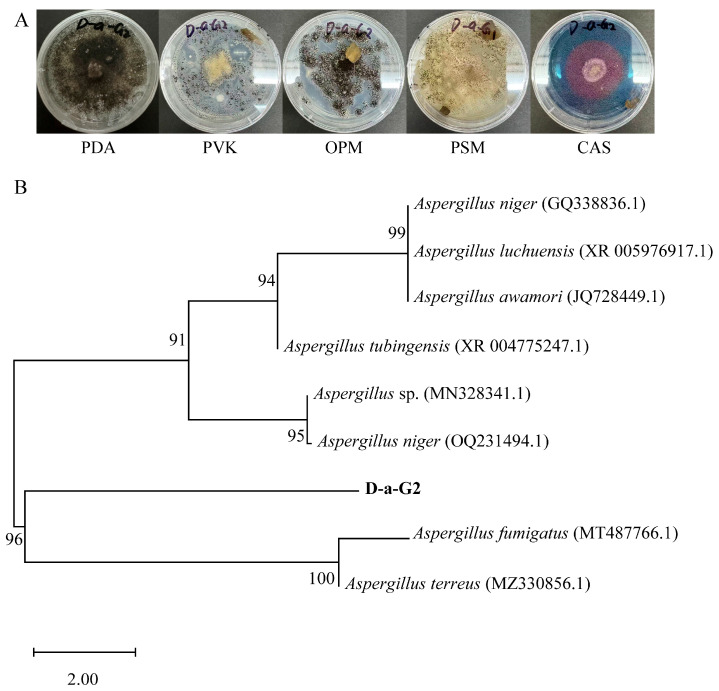
Morphological and molecular identification of the endophytic growth-promoting fungus isolated from *Nymphaea candida* roots. (**A**) Morphology of *Aspergillus* sp. on Potato Dextrose Agar (PDA), Pikovskaya’s Agar Medium (PVK), Phosphate-solubilizing bacteria medium (OPM), Potassium bacteria Medium (PSM), and Chrome Azurol S Agar Medium (CAS). (**B**) Neighbor-joining phylogenetic tree constructed based on the 18S rRNA gene sequence of the endophytic growth-promoting fungus *Aspergillus* sp. obtained from *Nymphaea candida* in this study. The strain numbers shown in bold represent the isolates obtained in this study. The species name indicates the molecularly identified species and D-a-G2 is the strain number. GenBank accession numbers are shown in parentheses after the strain names. The branching pattern was generated using the neighbor-joining method, bootstrap analysis was performed with 1000 replicates, and the scale bar represents 0.5 nucleotide substitutions per site.

Nine endophytic fungal strains were isolated from the roots of *N. candida* (Table S1). Among them, strain D-a-G2 exhibited black coloration, an uneven surface, irregular colony margins, relatively light-colored mycelia, and loose association with the culture medium. This strain solubilizes organic and inorganic phosphate, solubilizes potassium, and produce siderophores (Figure 1A). The results showed that the sequence similarity between strain D-a-G2 and *A. fumigatus* exceeded 99%, but based on the current data, it was identified only at the genus level (Figure 1B). Thus, this fungus isolated from the roots of *N. candida* was identified as *Aspergillus* sp.

### 3.2. Effects of Aspergillus sp. on Leaf and Root Growth of N. candida Seedlings

**Figure 2:**
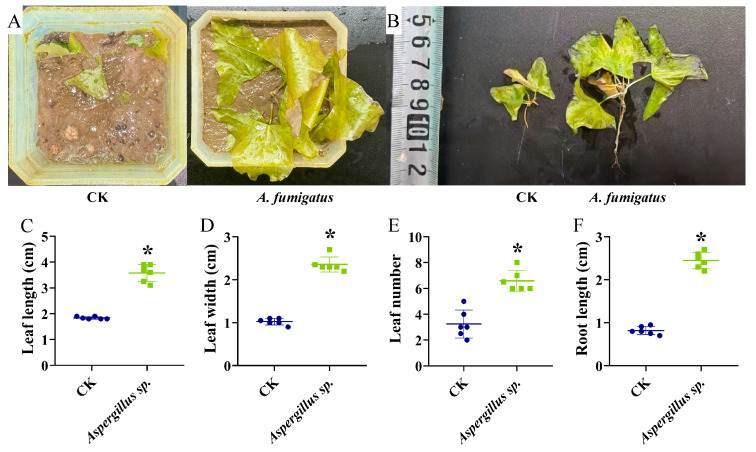
Effects of *Aspergillus* sp. on leaf morphology (**A**), root morphology (**B**), leaf length (**C**), leaf width (**D**), leaf number (**E**), and root length (**F**) of *Nymphaea candida* seedlings. All experimental groups had six replicates, and data are presented as mean ± standard deviation (*n* = 6). Statistical significance was indicated as follows: * *p* < 0.05. Statistical significance was determined by two-tailed Student’s *t*-test.

Compared with the control group, the inoculation of *Aspergillus* sp. for 60 days significantly increased leaf length, leaf width, and leaf number of *N. candida* seedlings by 97.83 ± 0.42%, 131.37 ± 0.36%, and 94.12 ± 2.45%, respectively (*p* < 0.05; Figure 2A,C–E). Notably, the root length of *N. candida* seedlings in the *Aspergillus* sp. group was 1.71-fold greater than that of the control group (*p* < 0.05; Figure 2B,F).

### 3.3. Effects of Aspergillus sp. on Soil Physicochemical Properties

**Figure 3:**
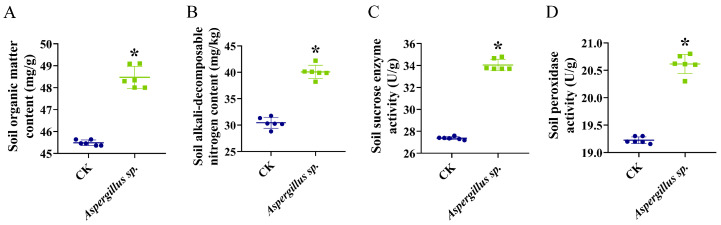
Effects of *Aspergillus* sp. on organic matter content (**A**), alkali-hydrolyzable nitrogen content (**B**), sucrase activity (**C**), and peroxidase activity (**D**) in the cultivation soil of *Nymphaea candida* seedlings. All experimental groups had six replicates, and data are presented as mean ± standard deviation (*n* = 6). Statistical significance was indicated as follows: * *p* < 0.05. Statistical significance was determined by two-tailed Student’s *t*-test.

After the application of *Aspergillus* sp., soil organic matter content increased from 45.49 ± 0.13 g/kg to 48.47 ± 0.50 g/kg, alkali-hydrolyzable nitrogen content increased from 30.44 ± 1.03 mg/kg to 40.07 ± 1.27 mg/kg, sucrase activity increased from 27.38 ± 0.13 U/g to 34.06 ± 0.52 U/g, and peroxidase activity increased from 19.22 ± 0.06 U/g to 20.61 ± 0.18 U/g (*p* < 0.05; Figure 3A–D).

### 3.4. Effects of Aspergillus sp. on Rhizosphere Prokaryotic Microbial Communities

**Figure 4:**
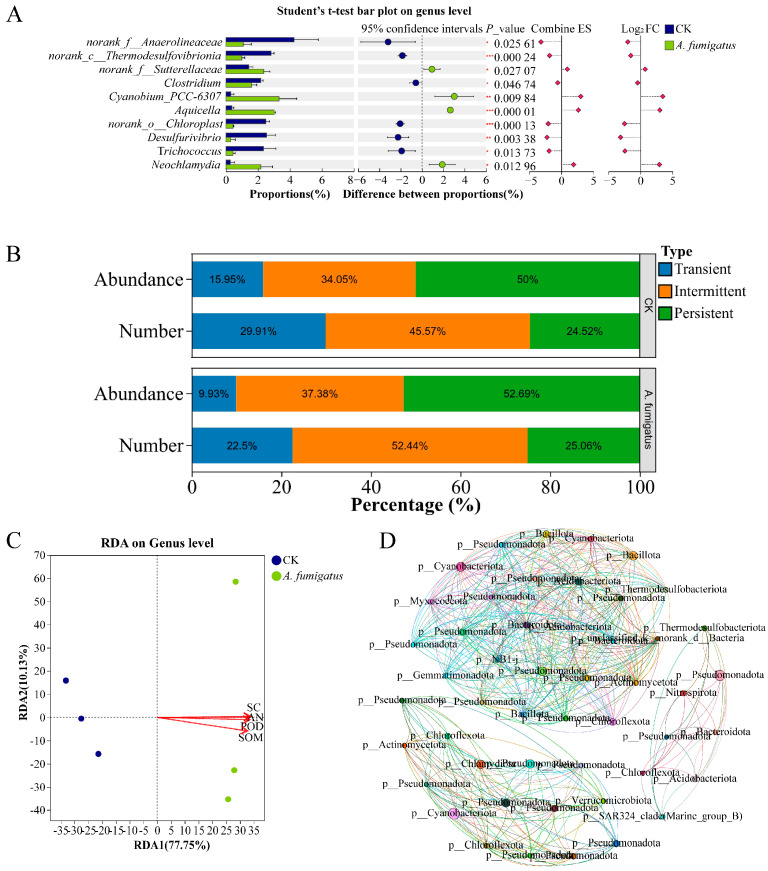
Effects of *Aspergillus* sp. on the composition and structure of prokaryotic microbial communities in the rhizosphere soil of *Nymphaea candida* seedlings. (**A**) Bar chart comparing the two groups showing significant differences in the mean relative abundance of the same taxonomic unit between different groups. Statistical significance is indicated as follows: * 0.01 < *p* < 0.05, ** 0.001 < *p* < 0.01, *** *p* < 0.001. (**B**) Environmental sensitivity of various types of prokaryotic microorganisms in the rhizosphere soil of *Nymphaea candida* from different experimental groups. (**C**) Response (RDA) analysis of the effects of key physicochemical indicators of the *Nymphaea candida* cultivation environment on rhizosphere prokaryotic microbial communities following *Aspergillus* sp. application. Soil organic matter (SOM), alkali-hydrolyzable nitrogen (AN), sucrase (SC), and peroxidase (POD). (**D**) Co-occurrence network analysis of prokaryotic microorganisms in the rhizosphere soil of the *Aspergillus* sp. group. Node colors were defined by different genera, and node sizes were defined by degree. Co-occurrence networks were constructed based on the relative abundances of prokaryotic microbial genera. Spearman’s correlation coefficients were calculated using the “psych” package (v 2.6.3) in R. Significant correlations (|r| > 0.6, *p* < 0.05) were used to build networks using the “igraph” package (v 2.2.3). Topological parameters (network density, modularity, and weighted average degree) were calculated to compare network complexity and stability between the control and *Aspergillus* sp. treatment groups.

The application of *Aspergillus* sp. had no significant effect on the alpha and beta diversity of prokaryotic microorganisms in the rhizosphere soil of *N. candida* (Figure S1A–D). RDA results indicated that, compared with the control group, the rhizosphere prokaryotic microbial community in the *Aspergillus* sp. group was significantly positively correlated with soil sucrase activity (r^2^ = 0.99, *p* < 0.05; Figure 4C). Moreover, the relative abundances of *Cyanobium*-PCC-6307, *Aquicella*, and *Neochlamydia* in the *Aspergillus* sp. group significantly increased by 10.18, 7.08, and 6.78-fold, respectively (*p* < 0.05; Figure 4A). Meanwhile, the relative abundances of intermediate and persistent prokaryotic microbial communities significantly increased to 37.38% and 52.69%, respectively (Figure 4B). The application of *Aspergillus* sp. also increased the network density, modularity, and weighted average degree of the prokaryotic microbial co-occurrence network in the *N. candida* rhizosphere by 18.65%, 34.15%, and 42.73%, respectively (Figure 4D, Table S2).

### 3.5. Effects of Aspergillus sp. on Rhizosphere Eukaryotic Microbial Communities

**Figure 5:**
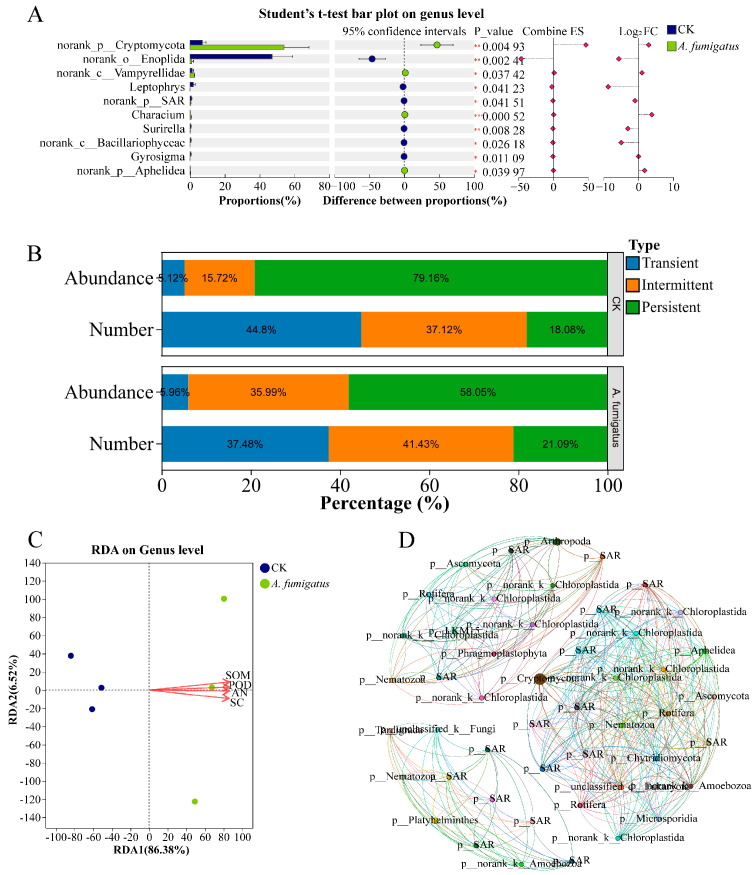
Effects of *Aspergillus* sp. on the composition and structure of eukaryotic microbial communities in the rhizosphere soil of *Nymphaea candida* seedlings. (**A**) Bar chart comparing the two groups showing significant differences in the mean relative abundance of the same taxonomic unit between different groups. Statistical significance is indicated as follows: * 0.01 < *p* < 0.05, ** 0.001 < *p* < 0.01, *** *p* < 0.001. (**B**) Environmental sensitivity of various types of eukaryotic microorganisms in the rhizosphere soil of *Nymphaea candida* from different experimental groups. (**C**) Response (RDA) analysis of the effects of key physicochemical indicators of the *Nymphaea candida* cultivation environment on rhizosphere eukaryotic microbial communities following *Aspergillus* sp. application. Soil organic matter (SOM), alkali-hydrolyzable nitrogen (AN), sucrase (SC), and peroxidase (POD). (**D**) Co-occurrence network analysis of eukaryotic microorganisms in the rhizosphere soil of the *Aspergillus* sp. group. Node colors were defined by different genera, and node sizes were defined by degree. Co-occurrence networks were constructed based on the relative abundances of eukaryotic microbial genera. Spearman’s correlation coefficients were calculated using the “psych” package in R. Significant correlations (|r| > 0.6, FDR-adjusted *p* < 0.05) were used to build networks using the “igraph” package. Topological parameters (network density, modularity, and weighted average degree) were calculated to compare network complexity and stability between the control and *A. fumigatus* treatment groups.

The application of *Aspergillus* sp. had no significant effect on alpha and beta diversity of eukaryotic microorganisms in the rhizosphere soil of *N. candida* (Figure S1C,D). RDA results indicated that, compared with the control group, the structure of the rhizosphere eukaryotic microbial community in the *Aspergillus* sp. group was positively correlated with soil organic matter content (r^2^ = 0.98, *p* < 0.05), alkali-hydrolyzable nitrogen content (r^2^ = 0.98, *p* < 0.05), sucrase activity (r^2^ = 0.98, *p* < 0.05), and peroxidase activity (r^2^ = 0.98, *p* < 0.05; Figure 5C). *Aspergillus* sp. significantly increased the relative abundances of norank-p-*Cryptomycota*, norank-c-*Vampyrellidae*, and *Characium* by 6.61, 1.01, and 13.19-fold, respectively (*p* < 0.05; Figure 5A). After the application of *Aspergillus* sp., the relative abundance of transient and intermediate eukaryotic microbial communities significantly increased to 5.96% and 35.99%, respectively (Figure 5B). Moreover, the application of *Aspergillus* sp. increased the network density, modularity, and weighted average degree of the eukaryotic microbial co-occurrence network in the *N. candida* rhizosphere by 4.19%, 2.02%, and 3.61%, respectively (Figure 5D; Table S2).

### 3.6. Effects of Aspergillus sp. on Gene Expression in N. candida Leaves

**Figure 6:**
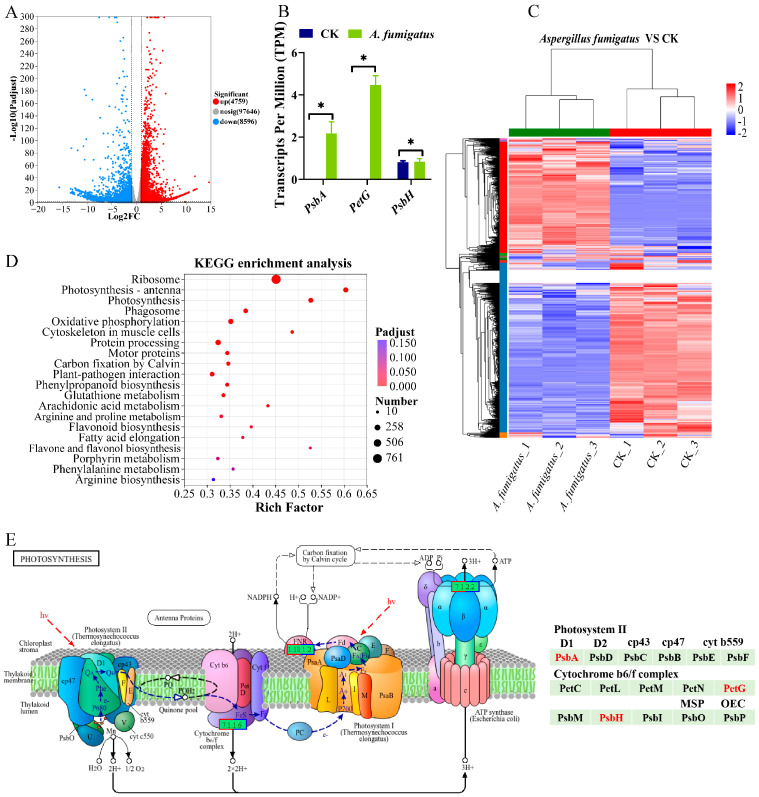
Effects of *Aspergillus* sp. on gene expression in the transcriptome of *Nymphaea candida* leaves. (**A**) Volcano plot showing the distribution of differentially expressed genes in *Nymphaea candida* leaves from the *Aspergillus* sp. Group. (**B**) Relative quantification of upregulated genes in the photosynthesis pathway (*psbA*, *petG*, *psbH*) of the *Aspergillus* sp. group compared to the control group. Statistical significance is indicated as follows: * *p* < 0.05, (**C**) Clustering heatmap of differentially expressed genes in the leaf transcriptome of *Nymphaea candida*. (**D**) Top 20 KEGG enrichment pathways in the *Aspergillus* sp. group compared to the control group. (**E**) KEGG reference photosynthesis pathway map onto which the differentially expressed genes identified in *N. candida* leaves were mapped. Genes with significant upregulation (*psbA*, *petG*, *psbH*) are highlighted in red.

Compared with the control group, 4759 genes were upregulated and 8956 genes were downregulated in the leaves of *N. candida* from the *Aspergillus* sp. group (Figure 6A). Clustering analysis results showed that the unigenes from the CK and *Aspergillus* sp. groups clustered independently into two separate groups, Cluster I and Cluster II, indicating distinct gene expression profiles between the two treatments. High reproducibility was observed among parallel samples, indicating that the data were suitable for further analysis (Figure 6C). KEGG pathway enrichment analysis revealed that the most significantly enriched metabolic pathways in the *Aspergillus* sp. group mainly included “Ribosome”, “Photosynthesis-antenna proteins”, and “Photosynthesis” (Figure 6D). Analysis of DEGs showed that after *Aspergillus* sp. application, the transcripts per million (TPM) value of the gene encoding the photosystem II P680 reaction center D1 protein (*psbA*) in the photosynthesis pathway was significantly upregulated by 2.17-fold, and the TPM value of the gene encoding cytochrome b6-f complex subunit 5 (*petG*) significantly increased by 4.48-fold. The TPM value of the gene encoding the photosystem II PsbH protein (*psbH*) also significantly increased by 28.4% (*p* < 0.05; Figure 6B,E).

## 4. Discussion

In this study, an endophytic growth-promoting fungus, *Aspergillus* sp., was isolated from the roots of *N. candida* and demonstrated the ability to produce siderophores, solubilizing organic and inorganic phosphate, and solubilizing potassium. Although 18S rRNA sequence similarity (>99%) and morphological characteristics suggest that strain D-a-G2 is closely related to *Aspergillus fumigatus*, we refer to it as *Aspergillus* sp. due to the lack of multi-locus phylogenetic analysis (e.g., benA, caM, rpb2). Future studies will employ additional loci or whole-genome sequencing for definitive species identification [36]. Moreover, the application of *Aspergillus* sp. significantly increased leaf length, leaf width, leaf number, and root length of *N. candida*, as well as the photosynthetic efficiency in the leaves. Thus, *Aspergillus* sp. could significantly enhance the growth of the rare and endangered aquatic plant *N. candida*.

The growth of aboveground and belowground plant parts is relatively coordinated, and the improvement in leaf traits might be attributed to the increased root length of *N. candida* [37]. Previous studies showed that *A. fumigatus* exerted growth-promoting effects through the production of plant growth hormones and biostimulants, secretion of organic acids or phosphatases, and induction of plant resistance, which was similar to arbuscular mycorrhizal fungi [38,39,40,41]. In this study, *Aspergillus* sp. significantly increased the root length of *N. candida* by 1.7-fold. The *Aspergillus nomius* WLG2 strain could also promoted root growth of maize via producing growth-promoting substances auxin and flavonoids, resulting in a 60% increase in maize root length [42]. The promoting effects of *A. fumigatus* on both the leaves and roots of *N. candida* seedlings provide direct evidence for its potential as an efficient endophytic growth-promoting fungus.

The growth-promoting effect of *Aspergillus* sp. on *N. candida* might be attributed to improvements in soil physicochemical properties. After inoculation with *Aspergillus* sp., the contents of organic matter and alkali-hydrolyzable nitrogen in the cultivation soil of *N. candida* seedlings significantly increased by 6.57% and 31.62%, respectively. Moreover, the sucrase and peroxidase activities increased by 23.26% and 7.53%, respectively. Soil enzyme activities are important indicators of soil biological activity and nutrient cycling capacity [43]. Sucrase participates in the decomposition of soil organic carbon and carbon cycling, and its increase indicated an enhanced mineralization rate of organic matter in the soil, potentially providing plants with more available carbon sources and nutrients [44]. Peroxidase is closely associated with the polymerization of soil organic matter and humus formation, and its increase contributed to the improvement of the soil structure [45]. Studies have shown that *A. fumigatus* strains solubilize phosphate, converting insoluble phosphorus in the soil into forms that are absorbable by plants, thereby significantly increasing root length in *Triticum aestivum* and *Vigna radiata* by 25% [46,47]. Thus, *Aspergillus* sp. might improve soil health through organic matter degradation and nutrient cycling promotion, thereby enhancing the growth of *N. candida* seedlings.

Although the diversity of the rhizosphere microbial community was unaffected, *Aspergillus* sp. enriched different beneficial microorganisms and formed a specific functional microbial community. The application of *Aspergillus* sp. significantly enriched *Cyanobium*-PCC-6307, *Aquicella*, and *Neochlamydia*. *Cyanobium*-PCC-6307 is a dominant cyanobacterial population in paddy soil [48], and *Aquicella* is a potential plant growth-promoting bacterium and might increase chlorophyll content and bulb number in onion by 6.46% and 21.41%, respectively [49]. *Neochlamydia*, an endosymbiont of *Vampyrellida*, exhibited increased abundance typically associated with population dynamics of amoebae in the environment, directly demonstrating the changes in the rhizosphere microbial community structure of *N. candida* [50]. Eukaryotic microbial community analysis revealed that the *Aspergillus* sp. group significantly enriched norank-p-*Cryptomycota*, norank-c-*Vampyrellidae*, and *Characium* in the rhizosphere of *N. candida*. Among these, *Cryptomycota* is a fungal group that participated in organic matter decomposition [51], while *Vampyrellidae* could prey on pathogenic fungi and algae to promote nutrient cycling and play a positive role in controlling soilborne diseases and maintaining environmental health [52]. *Characium*, as a photosynthetic autotroph, could utilize light energy to produce oxygen and organic matter and might serve as a marker for enhanced primary productivity in the ecosystems [53]. In this study, the application of *Aspergillus* sp. reduced the relative abundance of transient microorganisms, but increased the relative abundances of intermediate and persistent microbial communities, suggesting an enhanced adaptability of the rhizosphere microbial community to environmental changes [54]. RDA analysis results showed that the rhizosphere microbial community of *N. candida* was significantly positively correlated with organic matter content, alkali-hydrolyzable nitrogen content, sucrase activity, and peroxidase activity in the rhizosphere soil, further confirming that *Aspergillus* sp. indirectly promoted host growth by improving the soil microenvironment [55]. Co-occurrence network analysis demonstrated that the application of *Aspergillus* sp. markedly increased the modularity of both prokaryotic and eukaryotic microbial communities in the *N. candida* rhizosphere, resulting in a more complex network structure. Thus, *Aspergillus* sp. could form a more stable rhizosphere soil microenvironment to promote the growth of *N. candida* seedlings.

For the leaves, the application of *Aspergillus* sp. significantly upregulated genes encoding the photosystem II P680 reaction center D1 protein (*psbA*), cytochrome b6-f complex subunit 5 (*petG*), and photosystem II PsbH protein (*psbH*) in the photosynthetic metabolic pathway by 2.17, 0.28 and 4.48-fold, respectively. The enhanced photosynthesis enabled plants to generate more photosynthetic products and provide energy for all plant life activities [56]. Moreover, the abundant photosynthetic product reserves provided plants with more energy and materials for defense responses when facing exogenous stresses, and thereby promoted plant growth [57]. For instance, *A. oryzae* and *A. fumigatus* enhanced the photosynthesis in maize leaves and significantly increased root length by 11.76% [42]. Additionally, the enrichment of “Photosynthesis–antenna proteins” pathway suggested that *Aspergillus* sp. might promote photosynthesis by enhancing light energy capture efficiency, which corroborated the observed significant increase in leaf area of *N. candida* [58]. The upregulation of photosynthetic genes suggests that *Aspergillus* sp. may enhance photosynthetic capacity. Future studies will directly measure photosynthetic parameters (e.g., net photosynthetic rate, chlorophyll fluorescence) to confirm this inference. We acknowledge that the lack of independent qPCR validation for the RNA-seq data is a limitation of this study. Future studies will include RT-qPCR validation of key photosynthetic genes (*psbA*, *petG*, *psbH*) to confirm the transcriptomic findings.

## 5. Conclusions

In conclusion, the endophytic growth-promoting fungus *Aspergillus* sp. isolated from the roots of *N. candida* could promote leaf and root development of *N. candida* seedlings by increasing the contents of organic matter and alkali-hydrolyzable nitrogen in rhizosphere soil and enhancing sucrase and peroxidase activities. Moreover, the application of *Aspergillus* sp. enriched beneficial microorganisms including *Cyanobium*, *Aquicella*, and *Cryptomycota* to form a more stable rhizosphere soil microenvironment to promote the growth of *N. candida* seedlings. Furthermore, *Aspergillus* sp. upregulated the genes involved in photosynthesis and photosynthesis-antenna protein pathways in *N. candida* leaves to improve light energy utilization efficiency. Thus, *Aspergillus* sp. could be utilized as a potential biofertilizer for the propagation of *N. candida* seedlings, which would be helpful for the conservation of this rare and endangered aquatic plant species.

## Data Availability

The original contributions presented in this study are included in the article/Supplementary Material. Further inquiries can be directed to the corresponding authors.

## Supplementary Materials

The following supporting information can be downloaded at: https://www.mdpi.com/article/10.3390/microorganisms14050993/s1. Table S1. Sequence alignment result of the endophytic fungi isolated from the roots of *Nymphaea candida*; Table S2. Rhizosphere microbial network indices of *Nymphaea candida*; Figure S1: Alpha diversity of prokaryotic and eukaryotic microbial communities in the rhizosphere soil of *Nymphaea candida* (A,B); Principal coordinate analysis (PCoA) of prokaryotic and eukaryotic microbial communities based on Bray-Curtis distance (C,D).
