# Supplementary material for: Isolation and Identification of an Endophytic Fungus Aspergillus sp. and Its Growth-Promoting Effects on Nymphaea candida Seedlings Through Modulation of the Rhizosphere Microbial Community
Source: Microorganisms. 2026 Apr 28;14(5):993. doi: 10.3390/microorganisms14050993 (PMC13209681; doi:10.3390/microorganisms14050993)
Supplement: Supplementary file 1 [file microorganisms-14-00993-s001.zip › microorganisms-4239144-supplementary.pdf]

Supplementary Materials

Isolation and Identification of an Endophytic Fungus *Aspergillus* sp. and Its Growth-Promoting Effects on *Nymphaea candida* Seedlings Through Modulation of the Rhizosphere Microbial Community

Yuwei Xing, Jingru Zhang, Cong Liu, Yang Liu * and Jun Wang *

College of Marine Life Sciences, Ocean University of China, Qingdao 266003, China
* Correspondence: liuyang09@ouc.edu.cn (Y.L.); wangjun@ouc.edu.cn (J.W.);
Tel./Fax: +86-532-82031962 (J.W.)

Isolation and identification of endophytic fungi from the roots of Nymphaea candida

In this study, a total of 9 endophytic fungal strains were isolated from the roots of *Nymphaea candida*. Among them, 5 strains (B-A-d2, G-A-b1, L-A-b2, B-D2 and B-ba2) belonged to the phylum Ascomycota, 2 strains (D-a-G2 and B-ba3) belonged to the phylum Deuteromycotina, and 1 strain (B-D-d1) belonged to the phylum Heterokontophyta (Table S1).

Table S1. Sequence alignment result of the endophytic fungi isolated from the roots of *Nymphaea candida*.

Number	Strain	Taxon	Similarity (%)	Closest strain accession No.
B-A-d2	*Alternaria* sp.	Ascomycota	99.92%	MT649542.
G-A-b1	*Chaetomium globosum*	Ascomycota	99.77%	JQ964323.1
L-A-b2	Alternaria sp. SPS-04	Ascomycota	99.55%	KM250374.1
D-a-G2	*Aspergillus* sp.	Deuteromycotina	99.55%	MT487766.1
B-D-d1	*Phytopythium helicoides*	Heterokontophyta	99.78%	AY598665.2
G-A-11	fungal sp.	-	99.70%	KT714178.1
B-D2	*Fusarium venenatum*	Ascomycota	99.63%	LN649232.1
B-ba2	*Fusarium venenatum*	Ascomycota	99.62%	LN649232.1
B-ba3	*Trichoderma* sp. 2F	Deuteromycotina	99.85%	FJ716243.1

Effects of Aspergillus sp. application on alpha and beta diversity of rhizosphere microbial communities of N. candida

The application of *Aspergillus* sp. had no significant effect on the alpha and beta diversity of either the prokaryotic or eukaryotic microbial communities in the rhizosphere of *N. candida* seedlings (Fig. S1A–D).

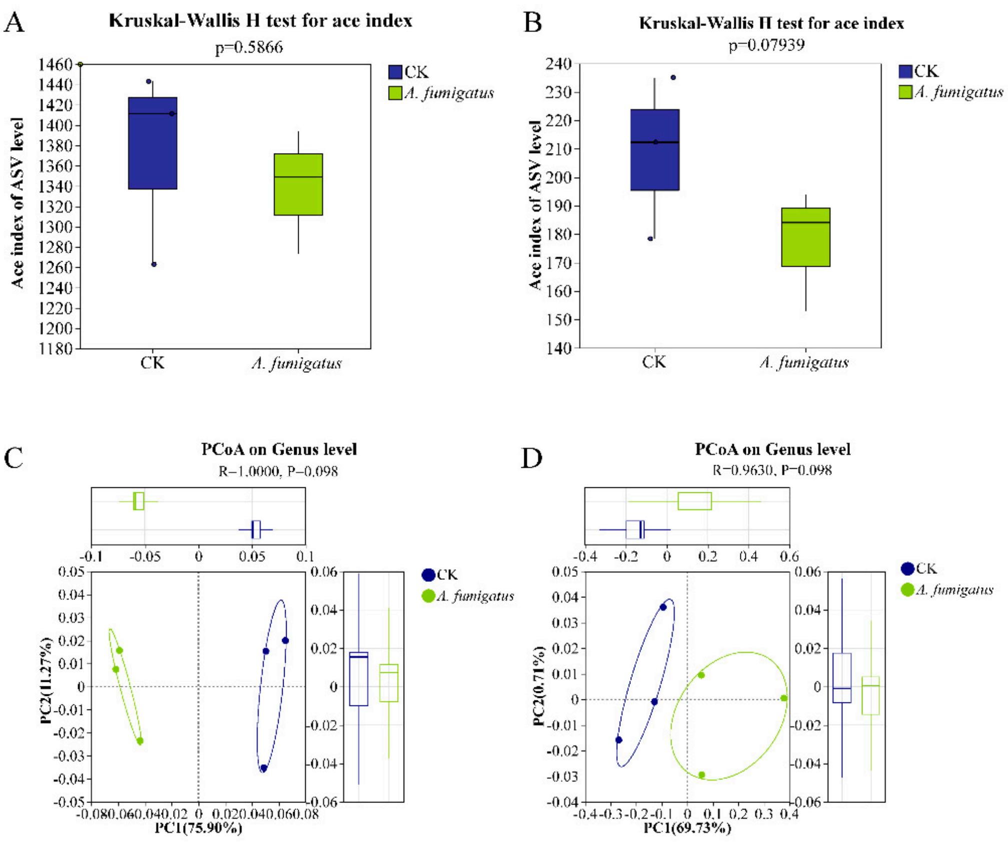

Figure S1. Effects of *Aspergillus* sp. application on alpha and beta diversity of rhizosphere microbial communities of *Nymphaea candida* (A–D). (A and B) Alpha diversity of prokaryotic and eukaryotic microbial communities in the rhizosphere soil of *Nymphaea candida*; (C and D) Principal coordinate analysis (PCoA) of prokaryotic and eukaryotic microbial communities based on Bray-Curtis distance.

Effects of Aspergillus sp. application on Co-occurrence networks of rhizosphere microbial communities of N. candida

The application of *Aspergillus* sp. also increased the network density, modularity, and weighted average degree of the prokaryotic microbial co-occurrence network in the *N. candida* rhizosphere by 18.65%, 34.15%, and 42.73%, respectively (Table S2). Moreover, the application of *Aspergillus* sp. increased the network density, modularity, and weighted average degree of the eukaryotic microbial co-occurrence network in the *N. candida* rhizosphere by 4.19%, 2.02%, and 3.61%, respectively (Table S2).

Table S2. Rhizosphere microbial network indices of *Nymphaea candida.*

Classification	Network density	Modularity	Weighted average degree
CK prokaryotic microorganisms	0.328	0.44	15.755
Aspergillus sp. prokaryotic microorganisms	0.389	0.628	18.694
CK eukaryotic microorganisms	0.347	0.527	15.957
Aspergillus sp. eukaryotic microorganisms	0.354	0.546	16.625
